# Use of Maternal-Fetal Medicine Subspecialist Services by Commercially Insured Pregnant People

**DOI:** 10.1001/jamanetworkopen.2024.54565

**Published:** 2025-01-13

**Authors:** Haley K. Sullivan, Joanne C. Armstrong, Kathe Fox, Jessica L. Cohen, Anna D. Sinaiko

**Affiliations:** 1 Harvard Interfaculty Initiative in Health Policy, Cambridge, Massachusetts; 2 Women’s Health and Genomics, CVS Health, Woonsocket, Rhode Island; 3 Health Care Cost Institute, Washington, DC; 4 Harvard School of Public Health, Boston, Massachusetts

## Abstract

**Importance:**

Improving access to high-quality maternity care and reducing maternal morbidity and mortality are major policy priorities in the US. Previous research has primarily focused on access to general obstetric care rather than access to high-risk pregnancy care provided by maternal-fetal medicine subspecialists (MFMs).

**Objective:**

To measure access to MFM services and determine patient factors associated with MFM service use, including MFM telemedicine.

**Design, Setting, and Participants:**

This cohort study identified pregnancies in commercial health insurance claims from the Health Care Cost Institute from 2016 to 2021. More than 2.1 million pregnancies were included, where age at delivery was 18 years or greater and people were continuously enrolled for the duration of their pregnancy. The association of patient and pregnancy covariates with MFM involvement in care was analyzed using logistic regression; and rates of telemedicine for pregnancies in urban and rural areas were reported over time. Data were analyzed from June 2022 to March 2024.

**Main Outcomes and Measures:**

Primary study outcomes included whether a pregnancy ever had a service from an MFM, the type of MFM services provided, and whether MFM care occurred via telemedicine.

**Results:**

There were 2 169 026 pregnancies among 1 968 091 unique people (1 325 212 [61.2%] aged 25 to 34 years). Among 1 625 237 pregnancies at risk for conditions that might require MFM involvement, 838 493 (51.6%) had an MFM service. Rates of MFM involvement in care varied considerably by geography, with pregnancies in rural areas having lower use than urban areas. Use of telemedicine-enabled MFM care increased in 2020 and 2021 but remained low: in 2021, 2.7% of urban pregnancies (7535 of 276 599) and 1.7% of rural pregnancies (550 of 32 949) received telemedicine-enabled MFM care.

**Conclusions and Relevance:**

In this cohort study, access to MFM services varied across geography, even among pregnancies at risk for conditions that might require MFM involvement. These results suggested a need to improve access to MFM care for at-risk pregnancies and to further explore expanded access via telemedicine.

## Key Points

**Question:**

How many commercially insured people in the US have access to maternal-fetal medicine subspecialist (MFM) services during pregnancy and what factors are associated with access?

**Findings:**

In this cohort study of more than 1.9 million pregnant people, access to MFM services varied across geography, even among pregnancies at risk for conditions that might require MFM involvement. Use of telemedicine-enabled MFM care increased in 2020 and 2021 but remained low; in 2021, 2.7% of urban and 1.7% of rural pregnancies received telemedicine-enabled MFM care.

**Meaning:**

These results suggest that policymakers should seek solutions to improve access to MFM care for at-risk pregnancies, including expanded access via telemedicine.

## Teaser

This cohort study measures access to maternal-fetal medicine (MFM) services and investigates factors associated with MFM service use, including MFM telemedicine.

## Introduction

Pregnant people in the US have higher risk of maternal morbidity and mortality than people in other high-income countries.^1,2^ Improving outcomes is a major policy priority—the Centers for Medicare and Medicaid Services recently announced a program to expand access to evidence-based maternal health care through a new care-delivery model.^3^ However, there are substantial gaps in access to critical aspects of maternity care. Existing research has primarily focused on the 39.8% of US counties without any general obstetricians, certified nurse midwives, maternity units within hospitals, or birthing centers—regions that the March of Dimes termed “maternity care deserts.”^1,4,5^ Although important, this research excludes a dimension of maternity care that can mitigate adverse outcomes: perinatal specialist care performed by maternal-fetal medicine subspecialists (MFMs).

MFMs are trained to manage complex pregnancies with the goal of reducing the risk of adverse delivery or newborn outcomes.^6^ The American College of Obstetricians and Gynecologists (ACOG) and the American Academy of Pediatrics recommend MFM consultation for pregnancies with known or suspected fetal abnormalities, severe existing disease that adversely affects pregnancy (eg, pregestational diabetes), and other conditions.^7^ MFMs also provide obstetrical ultrasound services and antenatal fetal surveillance (eg, biophysical profiles and nonstress tests).^6^ Referrals to, and subsequent care from, an MFM are associated with improved fetal outcomes, and lower cesarean delivery, preterm birth, and perinatal mortality rates.^8,9^

Previous research identified a higher density of MFMs in metropolitan counties^10^ and an inverse relationship between MFM density and maternal mortality rates.^11^ No research has quantified how this variation in MFM density relates to the number of people who receive care from MFMs in a national sample. There is a similar paucity of evidence surrounding telemedicine-enabled MFM care. Prior to the COVID-19 pandemic, when telemedicine use was limited,^12^ studies in Arkansas and Pittsburgh found telemedicine reduced distance traveled by people for MFM care and saved costs.^13,14^ Research during the COVID-19 pandemic reported high patient satisfaction with MFM telemedicine care; however, rates of telemedicine MFM usage since 2020 were not reported.^15,16,17^

We evaluate the utilization of MFM services in the US to inform policies designed to improve access to care and maternal health outcomes. Using a national, commercially insured sample of more than 2 million pregnancies from 2016 to 2021, we investigate individual-level factors associated with MFM involvement in care. Furthermore, we quantify the use of telemedicine-enabled MFM services before and during the COVID-19 pandemic to identify opportunities for improvement.

## Methods

This cohort study was deemed exempt by Harvard University institutional review board and did not require informed consent because it was deemed not to be human participants research. This study followed the Strengthening the Reporting of Observational Studies in Epidemiology (STROBE) reporting guideline.

### Data and Sample

We used Health Care Cost Institute (HCCI) data, which includes multipayer commercial health insurance claims (including fully insured and self-insured) for more than 55 million members per year (one-third of the US employer-sponsored insurance population). Using a previously published algorithm, we selected all pregnancies in people aged 18 to 54 years of at least 20 weeks of gestation that ended in stillbirth or live birth between January 1, 2016, and December 31, 2021 (eMethods 1 in Supplement 1).^18,19,20^ The start of the last menstrual period (LMP) was calculated for all pregnancies using gestational age at delivery and delivery date derived from the claims data. We defined pregnancy episodes (hereafter, pregnancy) as the period from the LMP to delivery. To observe all care during pregnancy and measure key study variables, we excluded people not continuously enrolled in a health plan in the HCCI dataset between their LMP and date of delivery and people with missing zip codes (eTable 1 in Supplement 1).

### Outcomes

Our primary study outcomes were whether a pregnancy had a service provided by an MFM clinician (MFM involvement), the type of MFM services provided, and whether MFM care occurred via telemedicine. For each pregnancy, we measured MFM involvement in care if the servicing provider (clinician or clinical practice) on any paid claim had a taxonomy code of 207VM0101X (maternal and fetal medicine). We used these claims as a measure of access, recognizing that there could be instances where MFMs exist in a geographic region, but patients experience barriers to access. We classified MFM services as evaluation and management visits (EM visits), ultrasounds, antenatal fetal surveillance (AFS), and delivery services using *Current Procedural Terminology (CPT)* or Healthcare Common Procedure Coding System (HCPCS) codes. Whether a service was provided via telemedicine was measured using *International Statistical Classification of Diseases and Related Health Problems, Tenth Revision (ICD-10)*, *CPT*, and place-of-service codes^21^ (eTable 2 in Supplement 1).

### Covariates

We measured pregnant person age at delivery (age) in 4 groups reported directly in the data: 18 to 24, 25 to 34, 35 to 44, or 45 to 54 years of age. We measured whether each person had 1 or more than 1 pregnancy that met study inclusion criteria (number of sample pregnancies). To measure whether a pregnancy was at risk and potentially warranted MFM involvement in care, we used the Antenatal and Neonatal Guidelines, Education, and Learning Systems (ANGELS) measure developed for this purpose; *ICD-9* diagnosis codes were cross-walked to *ICD-10* codes^22^ (eTable 3 in Supplement 1). This measure identified conditions from the prepregnancy period (eg, pregestational diabetes) and those that developed during pregnancy (eg, preeclampsia). We referred to pregnancies without any of these conditions as not at risk. To test the sensitivity of our results to the pregnancy risk measure, we conducted additional analyses using 2 measures that more narrowly define at-risk pregnancies: Agency for Healthcare Research and Quality’s (AHRQ) Availability of Outpatient Maternal Fetal Medicine and Specialty Care for Women With High Risk Pregnancies measure and ACOG’s indications for outpatient antenatal fetal surveillance.^23,24^

We classified pregnancies as urban or rural using zip code and Rural Urban Commuting Area Codes (eMethods 2 in Supplement 1).^25^ We measured county-level sociodemographic factors using the Centers for Disease Control and Prevention Social Vulnerability Index (SVI), which assigns counties to a percentile (ie, 0 to 0.99) measure of potential susceptibility to community-level stressors in 4 categories: socioeconomic status, household characteristics, racial and ethnic minority status, and housing type and transportation; higher indicates greater susceptibility.^26^ In sensitivity analyses, we used alternate area-level sociodemographic measures based on US census data (eMethods 2 in Supplement 1).

Finally, we measured driving distance from the center of each pregnant person’s zip code to the nearest MFM using MFM addresses in the National Plan and Provider Enumeration System (NPPES). We categorized driving distances as less than or equal to 20 miles, 21 to 60 miles, or at least 60 miles from an MFM (eMethods 2 in Supplement 1).

### Statistical Analysis

We reported the proportion of MFMs services that were EM visits, ultrasounds, AFS, deliveries, or other services, overall and by pregnancy risk. We analyzed the association of at-risk status, age, driving distance, number of sample pregnancies, and each SVI category with MFM involvement in care using logistic regression. The model included state and year fixed effects and clustered standard errors at the state level. We conducted sensitivity analyses with alternate measures of covariates (ie, urban/rural residency instead of driving distance,^24^ each of the alternate measures of at-risk status, and alternate measures of area-level income, race, and education).

To explore geographic variation in access to MFM care for at-risk pregnancies, we estimated the percentage of at-risk pregnancies per hospital service area (HSA) with MFM involvement in care using logistic regression. Models adjusted for differences in patient and area-level characteristics that affect MFM involvement in care, including age, number of sample pregnancies, SVI categories, and HSA and year fixed effects. Standard errors were clustered at the HSA level. Results are predicted probabilities based on these models and are presented as a map. To comply with HCCI privacy requirements, HSAs with fewer than 11 pregnancies were excluded. We calculated the percentage of pregnancies with MFM involvement in care that was provided in person, both in person and via telemedicine, or only via telemedicine, by year and by rural vs urban area. Data analysis was performed from June 2022 to March 2024 using R version 4.3.2 (R Foundation for Statistical Computing). Two-sided *P* < .05 was considered statistically significant.

## Results

### Sample Demographics

The study sample included 2 169 026 pregnancies for 1 968 091 unique people (1 325 212 [61.2%] aged 25 to 34 years) (Table); 1 924 870 pregnancies (88.7%) were in urban areas and 244 108 (11.3%) were in rural areas (eTable 4 in Supplement 1). Among rural pregnancies, 4.0% of patients (9726 of 244 111) were 20 or fewer miles, 56.6% (138 180 of 244 111) between 21 and 60 miles, and 39.4% (96 205 of 244 111) over 60 miles driving distance to the nearest MFM.

**Table.** Sample Demographics by Pregnancy Risk Level^a^

Characteristic	No. (%)		
	Overall (N = 2 169 026)	Not at-risk pregnancies (n = 543 789)	At-risk pregnancies (n = 1 625 237)
MFM involvement in care during pregnancy			
≥1 MFM claim	971 377 (44.8)	132 884 (24.4)	838 493 (51.6)
No MFM claims	1 197 649 (55.2)	410 905 (75.6)	786 744 (48.4)
No. of at-risk conditions			
0	543 789 (25.1)	543 789 (100.0)	NA
1	619 382 (28.6)	NA	619 382 (38.1)
≥2	1 005 855 (46.4)	NA	1 005 855 (61.9)
Any MFM ultrasound			
No	1 237 692 (57.1)	415 737 (76.5)	821 955 (50.6)
Yes	931 334 (42.9)	128 052 (23.6)	803 282 (49.4)
Any MFM evaluation and management visit			
No	1 689 301 (77.9)	508 463 (93.5)	1 180 907 (72.7)
Yes	479 725 (22.1)	35 326 (6.5)	444 330 (27.3)
Any MFM antenatal fetal surveillance			
No	1 773 151 (81.8)	517 537 (95.2)	1 255 614 (77.3)
Yes	395 875 (18.3)	26 252 (4.8)	369 623 (22.7)
Any MFM delivery			
No	2 133 959 (98.4)	540 900 (99.5)	1 593 059 (98.0)
Yes	35 067 (1.6)	2889 (0.5)	32 178 (2.0)
Any MFM other service			
No	1 932 826 (89.1)	523 579 (96.3)	1 409 247 (86.7)
Yes	236 200 (10.9)	20 210 (3.7)	215 990 (13.3)
Age range, y			
18-24	260 177 (12.0)	71 302 (13.1)	188 867 (11.6)
25-34	1 325 212 (61.2)	353 582 (65.3)	971 611 (59.9)
35-44	566 696 (26.2)	113 933 (21.0)	452 754 (27.9)
≥45	11 858 (0.6)	2736 (0.5)	9122 (0.6)
Driving distance to the nearest MFM, miles			
≤20	1 563 387 (72.1)	371 419 (68.3)	1 191 945 (73.4)
21-60	450 731 (20.8)	124 061 (22.8)	326 659 (20.1)
≥60	154 722 (7.1)	48 262 (8.9)	106 458 (6.6)
Social Vulnerability Index, median (IQR)			
Socioeconomic status	0.51 (0.26-0.75)	0.50 (0.26-0.75)	0.51 (0.26-0.75)
Household characteristics	0.47 (0.24-0.68)	0.47 (0.25-0.68)	0.47 (0.24-0.67)
Racial and ethnic minority status	0.76 (0.58-0.90)	0.74 (0.56-0.89)	0.77 (0.58-0.90)
Housing type and transportation	0.65 (0.38-0.81)	0.64 (0.38-0.80)	0.65 (0.39-0.82)
Any telemedicine during pregnancy			
No	2 154 174 (99.3)	543 066 (99.9)	1 611 108 (99.1)
Yes	14 852 (0.7)	723 (0.1)	14 129 (0.9)

Abbreviations: MFM, maternal-fetal medicine subspecialist; NA, not applicable.

^a^ Missing values are not included in the table. MFM services identified using *Current Procedural Terminology (CPT)* codes listed in eTable 2 in Supplement 1. The other MFM service codes that were found in the largest number of pregnancies were: *CPT* 36415, 93325, 81002, 36416, 93976, 96040, 90471, 81003, 90715, and 59000. This includes fetal Doppler echocardiograms and duplex scans.

### Services Provided by MFMs

Among the 1 625 237 pregnancies assessed to be at risk (74.9% of the full sample), 838 493 (51.6%) had MFM involvement in care. Among 543 789 pregnancies not at risk (25.1% of the full sample), 132 884 (24.4%) had MFM involvement in care (Table).

Among 824 912 at-risk pregnancies with MFM involvement in care, 95.8% had an ultrasound, 53.0% had an EM service, 44.1% had AFS, 3.8% had delivery services, and 25.8% had other MFM services (eTable 5 in Supplement 1). Among 146 465 not-at-risk pregnancies with MFM involvement, 96.4% had an ultrasound, 26.6% had an EM service, 19.8% had an AFS, 2.2% had a delivery service, and 15.2% had other MFM services (eTable 5 in Supplement 1). For all pregnancies, the most common other services related to maternal testing and diagnostics.

### Factors Associated With Access to MFMs

**Figure 1.**
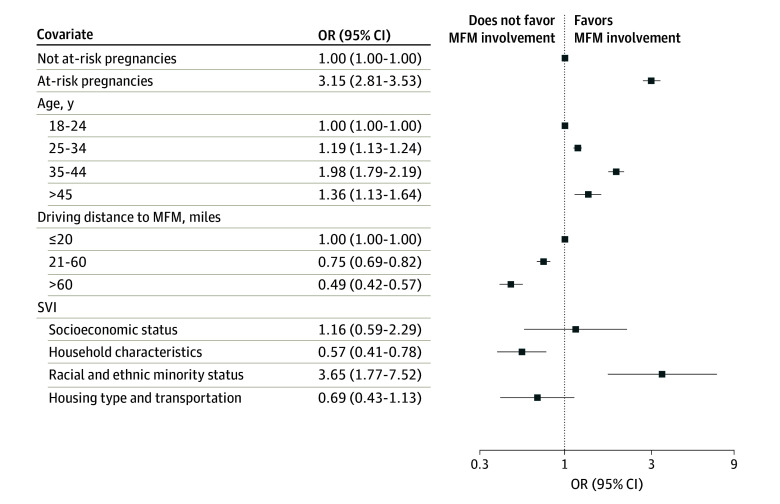
Odds of Maternal-Fetal Medicine Subspecialist (MFM) Involvement in Pregnancy Care Results show the odds ratios (ORs) and 95% CI for coefficients from logistic regression, adjusted for age, pregnancy risk, driving distance to nearest MFM, delivery year, pregnancy number in sample, and the Centers for Disease Control and Prevention Social Vulnerability Index (SVI). Model estimated on 2 163 687 pregnancies. SVI was reported with its 4 defined groups: socioeconomic status, household characteristics, racial and ethnic minority status, and housing type and transportation. Model includes state fixed effects, standard errors clustered at the state level.

In regression analysis, having an at-risk pregnancy was associated with a 3.15-fold increase in odds of MFM involvement in care (odds ratio [OR], 3.15 [95% CI, 2.81-3.53]) relative to a not-at-risk pregnancy (Figure 1). Living in an area with the highest susceptibility to community-level stressors for household characteristics was associated with decreased odds of MFM involvement in care by 43% (95% CI, 22%-59%) compared with living in an area with the least susceptibility. For a pregnancy moving from the 10th to 90th percentile of SVI for household characteristics, this was equivalent to a decreased probability of MFM involvement in care of 8.5 percentage points (23.5% effect). In contrast, living in an area with the highest susceptibility to community-level stressors for racial and ethnic minority status was associated with 3.65-fold higher odds of MFM involvement in care (OR, 3.65 [95% CI, 1.77-7.52]) compared with living in an area with the least susceptibility (Figure 1). For a pregnancy moving from the 10th to 90th percentile of SVI for racial and ethnic minority status, this was equivalent to an increased probability of having MFM involvement in care of 15.6 percentage points (66.0% effect). There was no significant association between MFM care and other SVI categories.

Compared with living within 20 miles of an MFM, living more than 60 miles from the nearest MFM was associated with 51% (95% CI, 44%-68%) lower odds of MFM involvement and living 21 to 60 miles away was associated with 25% lower odds (95% CI, 18%-31%) of MFM involvement in care (Figure 1).

Results from sensitivity analyses using alternate sociodemographic measures were similar. Using the AHRQ risk measure classified fewer (49.1%) pregnancies as at risk; however, 53.0% of the AHRQ at-risk pregnancies had MFM involvement in care, which is very similar to our main analysis (eTables 4 and 6 in Supplement 1). Living in a rural area was associated with a 22% (95% CI, 10%-33%) decrease in odds of MFM involvement in care compared with living in an urban area (eTable 6 in Supplement 1).

### Geographic Variation in MFM Involvement in Care of At-Risk Pregnancies

**Figure 2.**
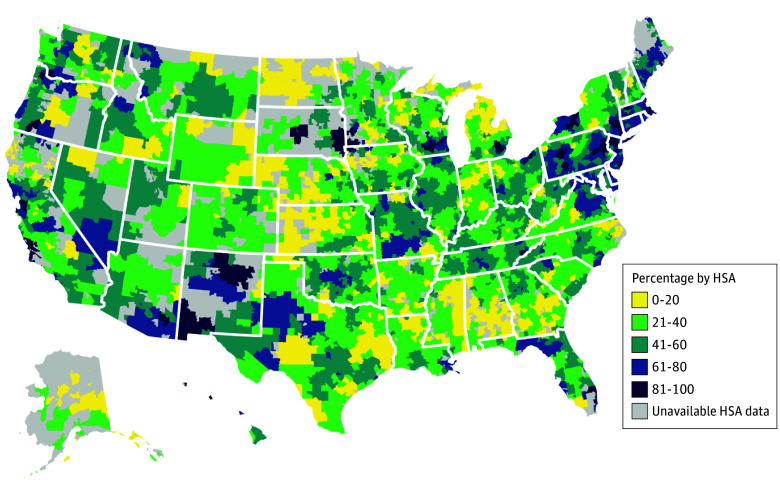
Predicted Probability of Maternal-Fetal Medicine Subspecialist (MFM) Involvement in Care of At-Risk Pregnancies by Hospital Service Area (HSA) Map of the US is color-coded to show the predicted probability of pregnancies with MFM service utilization by HSA. Model estimated on 1 625 237 pregnancies. Predicted probabilities from a logistic regression adjusted for age, pregnancy risk, driving distance to nearest MFM, delivery year, pregnancy number in sample, and Centers for Disease Control and Prevention Social Vulnerability Index. Model includes HSA fixed effects, standard errors clustered at the HSA level. HSAs with 10 or fewer pregnancies were excluded due to Health Care Cost Institute privacy requirements.

After adjusting for individual and pregnancy characteristics, the predicted probability of at-risk pregnancies with MFM involvement in care varied across HSAs from 2.5% to 99.3% (Figure 2). Generally, HSAs in the Northeast, in some regions of California, and in the Pacific Northwest had higher rates of MFM involvement in care when compared with most HSAs in the Midwest and South; some HSAs in New Mexico and South Dakota had comparably high access to care. Analysis of unadjusted geographic variation in MFM involvement in at-risk pregnancies and in the full sample were similar (eFigures 1 and 2 in Supplement 1).

### Trends in MFM Involvement and Telemedicine Over Time

**Figure 3.**
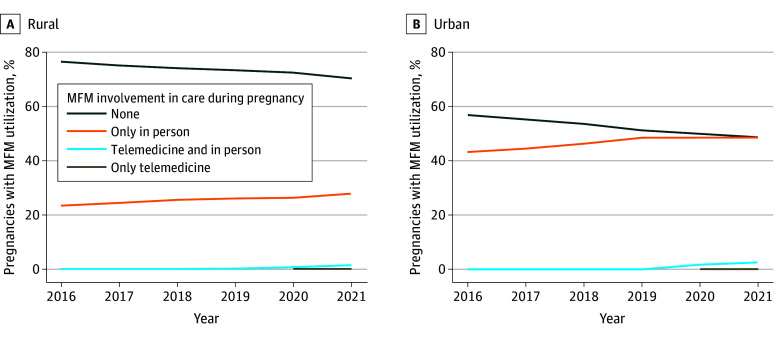
Comparison of In-Person and Telemedicine Maternal-Fetal Medicine Subspecialist (MFM) Utilization From 2016 to 2021 by Geographic Location Telemedicine use was identified via procedure codes, procedure code modifiers, and place-of-service codes (eTable 2 in Supplement 1). Rural or urban location was determined by the Rural Urban Commuting Area (RUCA) for the person’s zip code. RUCA codes were grouped into 2 standard categories: urban (codes 1.0, 1.1, 2.0, 2.1, 3.0, 4.1, 5.1, 7.1, 8.1, and 10.1), and rural (codes 4.0, 4.2, 5.0, 5.2, 6.0, 6.1, 7.0, 7.2, 7.3, 7.4, 8.0, 8.2, 8.3, 8.4, 9.0, 9.1, 9.2, 10.0, 10.2, 10.3, 10.4, 10.5, and 10.6). Values for only-telemedicine MFM visit during pregnancy were censored prior to 2020, when they did not meet the Health Care Cost Institute requirement for number of observations.

MFM involvement in care increased from 2016 to 2021 and was consistently higher in urban areas compared with rural areas. In 2016, 43.2% of urban residents (153 365 of 354 655) had MFM involvement in care compared with 23.5% of rural residents (10 984 of 46 824); in 2021, 51.3% of urban residents (142 036 of 276 599) had MFM involvement in care compared with 29.5% of rural residents (9725 of 32 949) (Figure 3). This corresponds to an increase of 8.1 percentage points for urban residents, and 6.1 percentage points for rural residents from 2016 to 2021.

Prior to the COVID-19 pandemic, fewer than 1% of all pregnancies had MFM care via telemedicine. This began to increase in 2020, and in 2021, 1.7% of rural pregnancies (550 of 32 949) and 2.7% of urban pregnancies (7535 of 276 599) had MFM care via telemedicine (Figure 3). Of 14 852 pregnancies with a telemedicine MFM service, 90.3% had a telemedicine MFM EM visit, 2.7% had telemedicine for an ultrasound, 0.9% had telemedicine for AFS, and 11.0% had another type of service (most commonly genetic counseling and nutrition therapy) (eTable 7 in Supplement 1).

## Discussion

In a commercially insured sample of more than 2.1 million pregnancies, 51.6% of at-risk pregnancies had any MFM involvement in care. Nearly all pregnancies with MFM involvement in care had MFM involvement in ultrasounds; MFMs were less likely to provide EM visits or other services. People living further from MFMs or in rural areas had significantly lower MFM involvement in pregnancy care. Rates of any MFM involvement increased from 2016 to 2021, by 8.1 percentage points for urban residents, and 6.1 percentage points for rural residents, but there was low use of telemedicine-enabled MFM care over the whole study period, which included the COVID-19 pandemic.

For many pregnancies, care from an MFM is clinically recommended and can potentially avert adverse pregnancy outcomes. In our sample, nearly half of at-risk pregnancies did not have any MFM involvement in care. To the best of our knowledge, this is the first published study that explores access to MFM services in general and at-risk obstetrical populations across the US. Prior studies largely relied on small sample sizes and limited geographic areas or used proxy variables for care utilization (eg, location of MFMs).^10,11,13,27^

The dominant MFM service provided to all pregnancies was obstetric ultrasound. At-risk pregnancies were more likely than not-at-risk pregnancies to have MFM involvement in EM visits, AFS, and delivery—suggesting that complex pregnancies are more likely to have MFM involvement in more complex services. However, our study cannot measure the appropriateness of MFM care; many factors influence service utilization including clinician network or payer reimbursement policy. Future research can assess whether utilization patterns represent overuse, underuse, or neither of MFM services.

Overall, access varied across the US, which may reflect availability of MFMs in selected areas of the country or geographic variation in practice patterns (eg, obstetricians in some regions may refer less frequently to MFMs, instead managing at-risk pregnancies on their own). Consistent with existing research that people living in rural areas have decreased access to specialist care,^28,29^ we found living in a rural area to be associated with lower rates of MFM involvement in pregnancy. Living 60 or more miles from an MFM was associated with lower odds of MFM involvement by nearly half compared with living 20 or fewer miles from an MFM. Exceptions to this pattern were observed in South Dakota and New Mexico, which had high rates of MFM involvement in care. Further research is needed to understand what factors increased access to MFMs in these areas.

Unlike distance, which had a strong association with access, associations between area-level measures of SVI and MFM involvement in care were mixed. Racial and ethnic minority status as per SVI categorization favored MFM involvement in care, whereas a higher SVI value for household characteristics did not favor MFM involvement.

Supplementing in-person MFM care with telemedicine, when appropriate, is a potential strategy to improve access to equitable perinatal care.^30^ The COVID-19 public health emergency (PHE) led to increased telemedicine in other services driven in part by new coverage and payment policies.^31^ In contrast, we found that MFM telemedicine was still uncommon. Potential barriers include licensing restrictions that prevent practice across state lines, malpractice concerns, startup costs, and state variation in private payer telehealth reimbursement, made more complicated by the end of the COVID-19 PHE and temporary payment policies.^31,32,33^ Recent efforts suggest these barriers are surmountable. For example, telemedicine, including MFM care, is a central component of the Rural Maternity and Obstetrics Management Strategies Program, which was implemented in 2021 and served more than 3100 people.^34,35^

### Limitations

There are limitations to this study. First, the categorization of at-risk pregnancies used a broad set of conditions that might benefit from MFM involvement identified with claims-based diagnoses codes and, as such, likely represents an upper bound of pregnancies that warranted MFM care. Sensitivity analyses using narrower measures to identify at-risk pregnancies classified fewer pregnancies as at-risk but found similar rates of pregnancies with MFM involvement. Second, claims data may not accurately reflect true clinical need for MFM care and although we discuss having an MFM claim as access to MFM care, we used utilization of services as an indirect measure of access. Third, measures of sociodemographic characteristics and driving distance describe an individual’s area of residence, rather than the individual themself. The measure of driving distance does not include whether the nearest MFM participates in a person’s insurance network; 20 miles driving may be prohibitive to care access in urban areas. Similarly, our measure of rurality may not reflect an individual’s perceptions of their own rural status. Fourth, our analysis is limited to the MFM services documented during the pregnancy period only.^19^ Although MFMs play important roles in the preconception and postpartum periods, these were out of scope for the present investigation. Furthermore, we required people to be continuously enrolled in their insurance plans throughout pregnancy to be included in the analysis. As such, these findings may not generalize to pregnancies where the person is changing, gaining, or losing insurance. Additionally, the HCCI data contains only commercial insurance claims and our findings may not generalize to the Medicaid population, which has lower income and constituted 41% of US births in 2021.^36^ We were not able to observe cost-sharing or any other plan-related factors that might affect access to MFM care.

## Conclusions

As policymakers and professional medical organizations seek to improve the maternity health crisis in the US, they should focus on access to MFM care given MFMs’ expertise in the management of complex pregnancies. Although rates of MFM involvement in pregnancy increased from 2016 to 2021, almost half of at-risk pregnancies in our analysis did not have MFM involvement in care. We identified wide geographic variation in MFM involvement in pregnancy care that is particularly acute in rural areas. Telemedicine is a potential solution to close these geographic gaps in access, but we found low rates of MFM telemedicine both before and during the COVID-19 pandemic. Exploration of current barriers and policies to support the expansion of safe and evidence-based MFM telemedicine services is needed.

## Notes

Supplement 1. **eMethods 1.** Methodology for Identifying Live Births and Stillbirths **eTable 1.** Sample Creation Table **eTable 2.** Codes for Classifying MFM Services by Type of Service and Telemedicine **eTable 3.** ANGELS Risk Measure ICD-10 Codes, Prevalence in Sample, and Rates of MFM Involvement in Care **eMethods 2.** Individual-Level Characteristics and Driving Distance **eTable 4.** Expanded Sample Characteristics **eTable 5.** MFM Services by Pregnancy Risk Status **eTable 6.** Regression Sensitivity Analyses **eFigure 1.** Unadjusted Probability of MFM Involvement in Care of At-Risk Pregnancies by Hospital Service Area (HSA) **eFigure 2.** Adjusted and Unadjusted Probability of MFM Involvement in Care in Full Sample by Hospital Service Area (HSA) **eTable 7.** Types of Telemedicine Services Provided by MFMs
eReferences

Supplement 2.
Data Sharing Statement

## References

[1] Brigance C, Lucas R, Jones E, . Nowhere to go: maternity care deserts across the U.S. (report No. 3). March of Dimes. 2022. Accessed December 2, 2024. https://www.marchofdimes.org/sites/default/files/2022-10/2022_Maternity_Care_Report.pdf

[2] World Health Organization. Trends in maternal mortality 2000 to 2017: estimates by WHO, UNICEF, UNFPA, World Bank Group and the United Nations Population Division. 2019. Accessed October 18, 2023. https://iris.who.int/handle/10665/327595

[3] CMS. HHS to improve maternal health outcomes with new CMS care model that expands access to services, other proven maternal health approaches. Accessed February 13, 2024. https://www.cms.gov/newsroom/press-releases/hhs-improve-maternal-health-outcomes-new-cms-care-model-expands-access-services-other-proven

[4] McGregor AJ, Hung P, Garman D, Amutah-Onukagha N, Cooper JA. Obstetrical unit closures and racial and ethnic differences in severe maternal morbidity in the state of New Jersey. Am J Obstet Gynecol MFM. 2021;3(6):100480. doi:10.1016/j.ajogmf.2021.100480 34496307

[5] Kozhimannil KB, Hung P, Henning-Smith C, Casey MM, Prasad S. Association between loss of hospital-based obstetric services and birth outcomes in rural counties in the United States. JAMA. 2018;319(12):1239-1247. doi:10.1001/jama.2018.1830 29522161 PMC5885848

[6] Sciscione A, Berghella V, Blackwell S, ; Society for Maternal-Fetal Medicine (SMFM). Society for maternal-fetal medicine (SMFM) special report: the maternal-fetal medicine subspecialists’ role within a health care system. Am J Obstet Gynecol. 2014;211(6):607-616. doi:10.1016/j.ajog.2014.09.013 25439812

[7] American Academy of Pediatrics, American College of Obstetricians and Gynecologists. Guidelines for Perinatal Care. Vol Appendix B, Appendix C. 8th ed. American College of Obstetricians and Gynecologists; 2017.

[8] Eden RD, Penka A, Britt DW, Landsberger EJ, Evans MI. Re-evaluating the role of the MFM specialist: lead, follow, or get out of the way. J Matern Fetal Neonatal Med. 2005;18(4):253-258. doi:10.1080/14767050500246292 16318976

[9] Givens M, Glover AV, Goodnight W, Menard MK, Manuck TA. 339: MFM-provided prenatal care is associated with a reduction in spontaneous PTB in high risk women. Am J Obstet Gynecol. 2019;220(1):S237. doi:10.1016/j.ajog.2018.11.360

[10] Rayburn WF, Klagholz JC, Elwell EC, Strunk AL. Maternal-fetal medicine workforce in the United States. Am J Perinatol. 2012;29(9):741-746. doi:10.1055/s-0032-1316445 22773289

[11] Sullivan SA, Hill EG, Newman RB, Menard MK. Maternal-fetal medicine specialist density is inversely associated with maternal mortality ratios. Am J Obstet Gynecol. 2005;193(3 Pt 2)(suppl):1083-1088. doi:10.1016/j.ajog.2005.05.085 16157116

[12] Shaver J. The state of telehealth before and after the COVID-19 pandemic. Prim Care. 2022;49(4):517-530. doi:10.1016/j.pop.2022.04.002 36357058 PMC9035352

[13] Magann EF, Bronstein J, McKelvey SS, Wendel P, Smith DM, Lowery CL. Evolving trends in maternal fetal medicine referrals in a rural state using telemedicine. Arch Gynecol Obstet. 2012;286(6):1383-1392. doi:10.1007/s00404-012-2465-5 22821508 PMC3880183

[14] Leighton C, Conroy M, Bilderback A, Kalocay W, Henderson JK, Simhan HN. Implementation and impact of a maternal-fetal medicine telemedicine program. Am J Perinatol. 2019;36(7):751-758. doi:10.1055/s-0038-1675158 30380582

[15] Jeganathan S, Prasannan L, Blitz MJ, Vohra N, Rochelson B, Meirowitz N. Adherence and acceptability of telehealth appointments for high-risk obstetrical patients during the coronavirus disease 2019 pandemic. Am J Obstet Gynecol MFM. 2020;2(4)(suppl):100233. doi:10.1016/j.ajogmf.2020.100233 32984803 PMC7506329

[16] Tozour JN, Bandremer S, Patberg E, . Application of telemedicine video visits in a maternal-fetal medicine practice at the epicenter of the COVID-19 pandemic. Am J Obstet Gynecol MFM. 2021;3(6):100469. doi:10.1016/j.ajogmf.2021.100469 34450341 PMC8454236

[17] Gillenwater JA, Rep MA, Troy AB, Power ML, Vigh RS, Mackeen AD. Patient perception of telemedicine in maternal-fetal medicine. Telemed J E Health. 2024;30(1):198-203. 37466478 10.1089/tmj.2023.0097

[18] Health Care Cost Institute. HCCI‘s 2.0 Commercial Claims Research Dataset. Published online 2021. Accessed December 2, 2024. https://healthcostinstitute.org/images//Health_Care_Cost_Institute_-_2dot0_Dataset_Overview_2021.pdf

[19] Ailes EC, Zhu W, Clark EA, . Identification of pregnancies and their outcomes in healthcare claims data, 2008-2019: an algorithm. PLoS One. 2023;18(4):e0284893. doi:10.1371/journal.pone.0284893 37093890 PMC10124843

[20] Geiger CK, Clapp MA, Cohen JL. Association of prenatal care services, maternal morbidity, and perinatal mortality with the advanced maternal age cutoff of 35 years. JAMA Health Forum. 2021;2(12):e214044. doi:10.1001/jamahealthforum.2021.4044 35977294 PMC8796879

[21] Hossain M, Dean EB, Kaliski D. Using administrative data to examine telemedicine usage among Medicaid beneficiaries during the coronavirus disease 2019 pandemic. Med Care. 2022;60(7):488-495. doi:10.1097/MLR.0000000000001723 35679172 PMC9172580

[22] Kozhimannil KB, Hung P, Casey MM, Lorch SA. Factors associated with high-risk rural women giving birth in non-NICU hospital settings. J Perinatol. 2016;36(7):510-515. doi:10.1038/jp.2016.8 26890556

[23] Agency for Healthcare Research and Quality. Availability of outpatient maternal fetal medicine and specialty care for women with high risk pregnancies. Accessed October 17, 2024. https://www.ahrq.gov/pqmp/measures/outpatient-speciality-care.html

[24] ACOG. Indications for outpatient antenatal fetal surveillance. Accessed October 30, 2023. https://www.acog.org/clinical/clinical-guidance/committee-opinion/articles/2021/06/indications-for-outpatient-antenatal-fetal-surveillance

[25] WWAMI Rural Health Research Center. Rural urban commuting area codes data. Accessed September 12, 2023. https://depts.washington.edu/uwruca/ruca-uses.php

[26] CDC/ATSDR SVI Data and Documentation Download | Place and Health | ATSDR. December 22, 2022. Accessed February 9, 2024. https://www.atsdr.cdc.gov/placeandhealth/svi/data_documentation_download.html

[27] Kroelinger CD, Brantley MD, Fuller TR, . Geographic access to critical care obstetrics for women of reproductive age by race and ethnicity. Am J Obstet Gynecol. 2021;224(3):304.e1-304.e11. doi:10.1016/j.ajog.2020.08.042 32835715 PMC9199012

[28] Brual J, Gravely-Witte S, Suskin N, Stewart DE, Macpherson A, Grace SL. Drive time to cardiac rehabilitation: at what point does it affect utilization? Int J Health Geogr. 2010;9(1):27. doi:10.1186/1476-072X-9-27 20525345 PMC2900239

[29] Polinski JM, Brookhart MA, Ayanian JZ, . Relationships between driving distance, rheumatoid arthritis diagnosis, and disease-modifying antirheumatic drug receipt. Arthritis Care Res (Hoboken). 2014;66(11):1634-1643. doi:10.1002/acr.22333 24664991 PMC4175286

[30] Healy A, Davidson C, Allbert J, Bauer S, Toner L, Combs CA; Society for Maternal-Fetal Medicine (SMFM). Electronic address: smfm@smfm.org; Patient Safety and Quality Committee. Society for Maternal-Fetal Medicine special statement: telemedicine in obstetrics-quality and safety considerations. Am J Obstet Gynecol. 2023;228(3):B8-B17. doi:10.1016/j.ajog.2022.12.002 36481188

[31] Mehrotra A, Wang B, Snyder G. Telemedicine: What Should the Post-Pandemic Regulatory and Payment Landscape Look Like? The Commonwealth Fund. 2020. Accessed December 3, 2024. https://www.commonwealthfund.org/sites/default/files/2020-08/Mehrotra_Medicare_Telemedicine_ib.pdf

[32] ACOG Committee Opinion. Implementing Telehealth in Practice; No 798. Obstet Gynecol. 2020;135(2). 31977794 10.1097/AOG.0000000000003672

[33] Laws ST, Report RP. Fall 2023. Center for Connected Health Policy. October 23, 2023. Accessed November 1, 2024. https://www.cchpca.org/resources/state-telehealth-laws-and-reimbursement-policies-report-fall-2023-2/

[34] U.S. Department of Health and Human Services, Health Resources and Services Administration, Federal Office of Rural Health Policy. Evaluation of the Rural Maternity and Obstetrics Management Strategies Program: first annual report. 2021. Accessed December 2, 2024. https://www.hrsa.gov/sites/default/files/hrsa/rural-health/2021-rmoms-annual-report.pdf

[35] U.S. Department of Health and Human Services, Health Resources and, Services Administration, Federal Office of Rural Health Policy. Rural Maternity and Obstetrics Management Strategies (RMOMS) Program. 2023. Accessed December 2, 2024. https://www.hrsa.gov/rural-health/grants/rural-community/rmoms

[36] Valenzuela CP, Osterman JKC. Products - Data Briefs - Number 468. Characteristics of mothers by source of payment for the delivery: United States, 2021. National Center for Health Statistics. May 2023. Accessed December 3, 2024. https://www.cdc.gov/nchs/data/databriefs/db468.pdf

