# Supplementary material for: Use of Maternal-Fetal Medicine Subspecialist Services by Commercially Insured Pregnant People
Source: JAMA Netw Open. 2025 Jan 13;8(1):e2454565. doi: 10.1001/jamanetworkopen.2024.54565 (PMC11731212; doi:10.1001/jamanetworkopen.2024.54565)
Supplement: Supplement 1. — eMethods 1. Methodology for Identifying Live Births and Stillbirths eTable 1. Sample Creation Table eTable 2. Codes for Classifying MFM Services by Type of Service and Telemedicine eTable 3. ANGELS Risk Measure ICD-10 Codes, Prevalence in Sample, and Rates of MFM Involvement in Care eMethods 2. Individual-Level Characteristics and Driving Distance eTable 4. Expanded Sample Characteristics eTable 5. MFM Services by Pregnancy Risk Status eTable 6. Regression Sensitivity Analyses eFigure 1. Unadjusted Probability of MFM Involvement in Care of At-Risk Pregnancies by Hospital Service Area (HSA) eFigure 2. Adjusted and Unadjusted Probability of MFM Involvement in Care in Full Sample by Hospital Service Area (HSA) eTable 7. Types of Telemedicine Services Provided by MFMs eReferences [file jamanetwopen-e2454565-s001.pdf]

Supplemental Online Content

Sullivan HK, Armstrong JC, Fox K, Cohen JL, Sinaiko AD. Use of maternal-fetal medicine subspecialist services by commercially insured pregnant people. *JAMA Netw Open.* 2025;8(1):e2454565. doi:10.1001/jamanetworkopen.2024.54565

eMethods 1. Methodology for Identifying Live Births and Stillbirths

eTable 1. Sample Creation Table

eTable 2. Codes for Classifying MFM Services by Type of Service and Telemedicine

eTable 3. ANGELS Risk Measure ICD-10 Codes, Prevalence in Sample, and Rates of MFM Involvement in Care

eMethods 2. Individual-Level Characteristics and Driving Distance

eTable 4. Expanded Sample Characteristics

eTable 5. MFM Services by Pregnancy Risk Status

eTable 6. Regression Sensitivity Analyses

eFigure 1. Unadjusted Probability of MFM Involvement in Care of At-Risk Pregnancies by Hospital Service Area (HSA)

eFigure 2. Adjusted and Unadjusted Probability of MFM Involvement in Care in Full Sample by Hospital Service Area (HSA)

eTable 7. Types of Telemedicine Services Provided by MFMs

eReferences

This supplemental material has been provided by the authors to give readers additional information about their work.

© 2025 Sullivan HK et al. *JAMA Network Open*.

eMethods 1: Methodology for Identifying Live Births and Stillbirths

We followed the algorithm developed by Ailes et al. (2023) to identify pregnancies in commercial claims data and to categorize the outcomes as live births, live birth and stillbirth in the same pregnancy, stillbirth, spontaneous abortion, elective abortion, or ectopic pregnancy.[1] The Ailes algorithm also estimated gestational age at the end of pregnancy using the latest gestational age diagnosis code available prior to delivery. We adjusted the spacing between pregnancies from 120 days to 30 days, due to pregnancy spacing observed in the data, and in line with other published pregnancy algorithms.[2,3] In the HCCI data, we do not have linked infant data, so we did not check our outcomes against infant data, as Ailes et al. do.

We removed any pregnancy outcomes that were not live births or stillbirths and adjusted the sequencing of pregnancies (e.g., first pregnancy of a person, second pregnancy, etc.) to reflect this. The Ailes et al. algorithm uses the estimated gestational age at delivery to compute a date for the first day of the Last Menstrual Period (LMP) before pregnancy. Finally, we required that all deliveries reach at least 20 weeks of gestation.

© 2025 Sullivan HK et al. *JAMA Network Open*.

eTable 1: Sample Creation Table

Stage of Analysis	Unique People in Sample (No.)	Percent Original Sample (%)	Percent Reduction (%)	Pregnancies in Sample (No.)	Percent Original Sample (%)	Percent Reduction (%)
People 18-54 years old with a pregnancy identified by the Ailes et al. (2023) algorithm	2,926,008	100%	--	3,385,703	100	--
Pregnancies that end in stillbirth or live birth and are 20+ weeks gestation at delivery	2,620,350	90%	10	2,903,710	86	14
Continuous enrollment from estimated Last Menstrual Period to the day of delivery	2,017,906	69%	23	2,225,247	66	23
Non-missing ZIP code	1,968,774	67%	2	2,169,719	64	2

NOTES: For the continuous enrollment requirement, we required that people be enrolled in a plan in the HCCI data from the calendar month of the LMP to the calendar month of delivery. Pregnancies could have a gap of 1 month in enrollment (except in the delivery month).

© 2025 Sullivan HK et al. *JAMA Network Open*.

eTable 2: Codes for Classifying MFM Services by Type of Service and Telemedicine

	CPT or HCPCS Code	CPT Code Modifiers	Place of Service Code
Radiology/Ultrasound[4]	76801, 76802, 76805, 76810, 76811, 76812, 76813, 76814, 76815, 76816, 76817		
Antenatal Fetal Surveillance[a4]	59020, 59025, 76818, 76819, 76820, 76821		
Delivery[4]	59400, 59409, 59510, 59514, 59610, 59612, 59618, 59620		
Evaluation and Management[4]	99201-99499		
Telemedicine Services[b5]	98966, 98967, 98968, 98970, 98971, 98972, 99091, 99421, 99422, 99423, 99441, 99442, 99443, 99457, 99458 G0406, G0407, G0408, G0425, G0426, G0427, G0508, G0509, G2010, G2012, G2061, G2062, G2063	GQ, GT, 95	02

[a] Nonstress test, Biophysical Profile, modified Biophysical Profile, or contraction stress test
[b] Some telemedicine services are also E&M services. We identified telemedicine services as either having one of the listed telemedicine-only CPT/HCPCS code or a CPT modifiers or Place of Service code for telemedicine.

© 2025 Sullivan HK et al. *JAMA Network Open*.

eTable 3: ANGELS Risk Measure ICD-10 Codes, Prevalence in Sample, and Rates of MFM Involvement in Care
Adapted from Kozhimannil et al. 2016[6]

	ICD-10 Diagnosis Codes	Percent of Sample with Diagnostic Code in a Given Category[c] (%)	Percent of Pregnancies with Condition with MFM Involvement (%)
**Chronic renal disease and other systemic diseases**	A40%, A41%, B15%, B16%, B17%, B18%, B19%, C50%, D72.89, D72.9, E00%, E01.8%, E03.0, E03.1, E03.2, E03.3, E03.8, E03.9, E05%, E89.0, K73%, K75.4, K81%, K82%, K85%, K86.0, K86.1, L93%, M32%, N03%, N05%, N06%, N07%, N08%, N17%, N18%, N19, N20%, N25%, N26.1, N26.9, N27%, O12.1%, O26.6%, O26.83%, O90.5, O98.6%, O99.28%	16.55	55.8
**HIV**	B20, Z21	0.10	70.12
**Herpes**	A60%, B0%	4.40	50.81
**Syphilis**	A50%, A51%, A52%, A53%, O98.1%	0.13	60.30
**Cervical Cancer**	C5%, D06%, N87%	0.62	51.92
**Uterine Abnormalities**	D25%, D26%, O34.0%, O34.1%, O34.4%, O34.5%, O34.6%, O34.7%, O34.8%, O34.9%	10.35	62.78
**Diabetes**	E10%, E11%, E13%, O24.0%, O24.1%, O24.3%, O24.8%, O24.9%	3.11	70.35
**Gestational Diabetes**	O24.4%	10.82	61.39
**Anemia**	D50%, O90.81, O99.0%	18.01	50.00
**Coagulation Disorders**	D65, D66, D67, D68.0%, D68.1%, D68.2%, D68.3%, D68.4%, D68.8, D68.9	0.59	69.16
**Hemorrhagic conditions**	D69%	2.20	55.75
**Epilepsy**	G40%, R56.9	0.64	59.37
**Cardiac Diseases**	I0%, I21%, I22%, I24%, I25.1%, I25.3%, I25.4%, I25.5, I25.6, I25.7%, I25.8%, I25.9%, I27%, I28%, I34%, I35.1%, I35.2%, I35.8%, I35.9%, I36.0%, I36.1%, I36.2%, I36.3%, I36.6%, I36.7%, I36.8%, I37%, I38%, I39%, I42%, I43%, I44%, I45%, I46%, I47%, I48%, I49%, I50% I51%, I97.0%, I97.1%, O99.4%, R00.1	3.90	62.29
**History of Cranial Injury**	I60%, I61%, I62%, I63%, I64%, I65%, I66%, I67.1, I67.2, I67.4, I67.5, I67.6, I67.7, I67.81, I67.82	0.14	65.95

[c] Percentages do not sum to 100% as a person might have more than one category of condition.

© 2025 Sullivan HK et al. *JAMA Network Open*.

**Pulmonary Diseases**	A15%, A17%, A18%, A19%, A22.1, A37.91, A48.1, B25,0, B44.0, B44.81, B44.9, J13%,J14%, J15%, J16%, J17, J18%, J39.8, J43%, J44.9, J47%, J6%, J70%, J80%, J81%, J82%, J84%, J85%, J86%, J90, J91%, J92%, J93%, J94%, J95.0%, J95.1, J95.2, J95.3, J95.811, J95.812, J95.821, J95.822, J85.84, J95.850, J96%, J98%, J99, M34.81, O98.0, R09.1, Z99.1	1.30	55.78
**Placental Bleeding**	O44.5%, O45%, O46.0%, O67.0%, O44.1%, O44.3%	2.44	58.93
**Hypertension, Pre-Eclampsia, Eclampsia***		19.29	52.24
Eclampsia	O15%	0.17	58.24
Essential Hypertension	I10%, I11%, I12%, I13%, I15%, I16%	3.47	61.95
Preeclampsia	O10%, O14%	10.66	58.81
Gestational Hypertensive Disorders	O11%, O13%, O16%	14.55	53.24
**Hyperemesis in Pregnancy**	O21%	8.45	47.54
**Recurrent Pregnancy Loss**	O26.2%	1.96	63.46
**Insufficient Cervix**	O34.3%	1.69	69.37
**Suspected fetal abnormalities and/or oligohydramnios, polyhydramnios, growth restriction**[d]		35.56	64.01
Known or Suspected Fetal Growth Restriction	O36.5%	12.17	54.85
Oligohydramnios	O41.0%	4.14	55.25
Polyhydramnios	O40%	3.70	65.15
Known or Suspected Fetal Abnormalities	O35.0%, O35.1%, O35.2%, O35.3%, O35.4%, O35.5%, O35.6%, O35.8%, O35.9%	23.24	74.07
**Previous Fetal Anomalies**	Excluded due to lack of ability to observe previous pregnancies	-	-
**Poor Obstetric History**	O09.21%, O09.29%	11.10	65.62

NOTES: In the ICD-10 Diagnosis Code column, the % sign is used to denote that all codes starting with that value were selected (e.g., O35.4% would select O35.41, O35.42, etc.)

[d] Patients may have more than one condition in these subcategories, therefore percents do not sum to 100.

© 2025 Sullivan HK et al. *JAMA Network Open*.

eMethods 2: Individual-Level Characteristics and Driving Distance

Geographic Location

Rural or urban geographic location was determined by the Rural Urban Commuting Area (RUCA) codes for the person's ZIP code. We categorized RUCA codes into 2 standard categories: urban (codes 1.0, 1.1, 2.0, 2.1, 3.0, 4.1, 5.1, 7.1, 8.1, and 10.1), and rural (4.0, 4.2, 5.0, 5.2, 6.0, 6.1, 7.0, 7.2, 7.3, 7.4, 8.0, 8.2, 8.3, 8.4, 9.0, 9.1, 9.2, 10.0, 10.2, 10.3, 10.4, 10.5, and 10.6).[7]

For people with more than one ZIP code during their pregnancy, we assigned them to the ZIP code where the person resided for the plurality of their pregnancy and, for ties, randomly selected one ZIP code.

Sociodemographic Characteristics

For the CDC Social Vulnerability Index (SVI), we cross-walked person ZIP code to county-level SVI measures of socioeconomic status, household composition and disability, minority status and language, and housing and transportation.[8]

As a sensitivity analysis, we proxied a person's race, income, and education with area-level measures associated with the person ZIP code from the 2015-2019 American Community Survey (ACS).[9] We classified all US ZIP codes into quintiles based on the percent of the population without a high-school degree and assigned each delivery to the education quintile of the person's ZIP code. A lower quintile corresponds to a higher percentage of people having a high school degree. We used ACS data to construct Indices of Concentration at the Extremes (ICE) measures of race and income. The ICE measure of race is calculated as the number of non-Hispanic white people in an area minus the number of Non-Hispanic Black people in the area divided by the total of those two groups. A lower quintile in this measure indicates a higher proportion of Black people in the ZIP code.[10] The ICE measure of income is calculated as the difference in the number of high-income people and low-income people in an area divided by the total number of people in the area with reported income; a higher quintile indicates a wealthier ZIP code.

Driving Distance

To calculate driving distance, we first obtained the address of the business practice location of an MFM as recorded in the NPPES database in 2021 (the final year of our study period). For each pregnancy, we determined if a person lived <=20 miles, 21-60 miles, or 60+ miles from an MFM by calculating if the centroid[11] of the person's ZIP code was contained within the 20- or 60-mile driving distances of an MFM.[12,13] We selected the 20-mile driving distance because it is the median distance that rural residents drive to obstetric hospital units.[14] Sixty miles is the threshold many state Medicaid programs use as the maximum distance acceptable for beneficiaries to drive to a specialist.[15] Although it would be ideal to use a continuous measure of driving distance, we selected these discrete thresholds to make the calculations computationally feasible.

© 2025 Sullivan HK et al. *JAMA Network Open*.

eTable 4: Expanded Sample Characteristics

Characteristic	**Overall**, N = 2,169,026 (100) No., (%)	**Not At-Risk Pregnancies**, N = 543,789 (25.1) No., (%)	**At-Risk Pregnancies**, N = 1,625,237 (74.9) No., (%)
**Sex**			
F	2,138,393 (98.59)	519,898 (95.61)	1,618,495 (99.59)
M	30,611 (1.41)	23,883 (4.39)	6,728 (0.41)
**Delivery Year**			
2016	401,478 (18.51)	118,762 (21.84)	282,716 (17.40)
2017	392,854 (18.11)	103,939 (19.11)	288,915 (17.78)
2018	367,548 (16.95)	91,941 (16.91)	275,607 (16.96)
2019	352,920 (16.27)	83,385 (15.33)	269,535 (16.58)
2020	344,660 (15.89)	80,026 (14.72)	264,634 (16.28)
2021	309,544 (14.27)	65,728 (12.09)	243,816 (15.00)
**Pregnancy Outcome**			
Any Stillbirth	20,563 (0.95)	4,037 (0.74)	16,526 (1.02)
Live Birth	2,148,441 (99.05)	539,744 (99.26)	1,608,697 (98.98)
**Gestational Age at Delivery: Median (IQR)**	39.00 (38.00, 39.00)	39.00 (39.00, 40.00)	39.00 (38.00, 39.00)
**ACOG Risk Measure**[16]			
Low Risk	659,393 (30.40)	343,500 (63.17)	315,893 (19.44)
High Risk	1,509,610 (69.60)	200,280 (36.83)	1,309,330 (80.56)
**AHRQ Risk Measure**[17]			
Not At-Risk	1,103,838 (50.89)	450,549 (82.85)	653,289 (40.20)
At-Risk	1,065,188 (49.11)	93,240 (17.15)	971,948 (59.80)
**RUCA**			
Rural	244,108 (11.25)	73,413 (13.50)	170,695 (10.50)
Urban	1,924,870 (88.75)	470,361 (86.50)	1,454,509 (89.50)
**FAR1**			
No	2,118,657 (97.68)	526,495 (96.82)	1,592,162 (97.96)
Yes	50,369 (2.32)	17,294 (3.18)	33,075 (2.04)
**Moved ZIP Codes During Pregnancy**	142,919 (6.59)	34,158 (6.28)	108,761 (6.69)
**Social Vulnerability Index: Median (IQR)**	0.59 (0.34, 0.79)	0.58 (0.33, 0.79)	0.59 (0.34, 0.79)
**Education Quintile**			
1	485,010 (22.36)	121,535 (22.35)	363,475 (22.36)
2	556,801 (25.67)	141,630 (26.05)	415,171 (25.55)
3	459,431 (21.18)	115,763 (21.29)	343,668 (21.15)
4	393,736 (18.15)	97,072 (17.85)	296,664 (18.25)
5	274,026 (12.63)	67,781 (12.46)	206,245 (12.69)
**ICE Income Quintile**			
1	121,391 (5.60)	28,486 (5.24)	92,905 (5.72)
2	363,074 (16.74)	95,522 (17.57)	267,552 (16.46)
3	477,765 (22.03)	126,247 (23.22)	351,518 (21.63)
4	596,457 (27.50)	150,717 (27.72)	445,740 (27.43)
5	610,291 (28.14)	142,800 (26.26)	467,491 (28.77)
**ICE Race Quintile**			
1	216,248 (9.97)	44,750 (8.23)	171,498 (10.55)

© 2025 Sullivan HK et al. *JAMA Network Open*.

2	489,252 (22.56)	115,145 (21.17)	374,107 (23.02)
3	603,749 (27.84)	151,962 (27.95)	451,787 (27.80)
4	536,472 (24.73)	142,067 (26.13)	394,405 (24.27)
5	323,283 (14.90)	89,857 (16.52)	233,426 (14.36)

NOTES: Gestational age at delivery is estimated as per eMethods 1. Rural or urban location was determined by the Rural Urban Commuting Area (RUCA) for the person's ZIP code. We categorized RUCA codes into 2 standard categories: urban (codes 1.0, 1.1, 2.0, 2.1, 3.0, 4.1, 5.1, 7.1, 8.1, and 10.1), and rural (4.0, 4.2, 5.0, 5.2, 6.0, 6.1, 7.0, 7.2, 7.3, 7.4, 8.0, 8.2, 8.3, 8.4, 9.0, 9.1, 9.2, 10.0, 10.2, 10.3, 10.4, 10.5, and 10.6).[7] ICE Income and Race Quintiles refer to Index of Concentration at the Extremes (ICE) measures as described in eMethods 2. The FAR1 measure describes "areas up to 50,000 people that are 60 minutes or more from an urban area of 50,000 or more people."[18]

© 2025 Sullivan HK et al. *JAMA Network Open*.

eTable 5: MFM Services by Pregnancy Risk Status

	**Overall**, N=971,377 (100) No., (%)	**Not At-Risk Pregnancies**, N=146,465 (15.1) No., (%)	**At-Risk Pregnancies,** N=824,912 (84.9) No., (%)
**Any MFM ultrasound**			
No	40,043 (4.1)	4,832 (3.6)	35,211 (4.2)
Yes	931,334 (96.4)	128,052 (96.4)	803,282 (95.8)
**Any MFM evaluation and management (E&M) visit**			
No	491,652 (50.6)	97,558 (73.4)	394,163 (47.0)
Yes	479,725 (49.4)	35,326 (26.6)	444,330 (53.0)
**Any MFM antenatal fetal surveillance**			
No	575,502 (59.2)	106,632 (80.2)	468,870 (55.9)
Yes	395,875 (40.8)	26,252 (19.8)	369,623 (44.1)
**Any MFM delivery**			
No	936,310 (96.4)	129,995 (97.8)	806,315 (96.2)
Yes	35,067 (3.6)	2,889 (2.2)	32,178 (3.8)
**Any MFM other service**			
No	735,177 (75.7)	112,674 (84.8)	622,503 (74.2)
Yes	236,200 (24.3)	20,210 (15.2)	215,990 (25.8)

NOTES: MFM services identified using CPT codes listed in eTable 2. The “other” MFM service codes that were found in the largest number of pregnancies were: CPT 36415, 93325, 81002, 36416, 93976, 96040, 90471, 81003, 90715, 59000. This includes fetal Doppler echocardiograms and duplex scans.

© 2025 Sullivan HK et al. *JAMA Network Open*.

eTable 6: Regression Sensitivity Analyses

	(1)	(2)	(3)	(4)	(5)
**ANGELS Risk**					
Not At-Risk	Ref			Ref	Ref
At-Risk	3.15*** (2.81,3.53)			3.15*** (2.82,3.52)	3.14*** (2.82,3.51)
**AHRQ Risk**[17]					
Not At-Risk		Ref			
At-Risk		2.00 *** (1.90, 2.12)			
**ACOG Risk**[16]					
Low			Ref		
High			2.47*** (2.27,2.68)		
**Driving Distance to Nearest MFM**					
<=20 Miles	Ref	Ref	Ref		Ref
21-60 miles	0.75*** (0.69,0.82)	0.75 *** (0.68, 0.82)	0.75*** (0.69,0.82)		0.68*** (0.62,0.75)
>60 Miles	0.49*** (0.42,0.57)	0.49 *** (0.42,0.57)	0.49*** (0.43,0.57)		0.43*** (0.37,0.49)
**RUCA**					
Urban				Ref	
Rural				0.78*** (0.67,0.90)	
**SVI**	X	X	X	X	
**ICE Quintiles**					X
**Other Covariates**	X	X	X	X	X
**State Fixed Effects**	X	X	X	X	X
Std.Errors	by: State	by: State	by: State	by: State	by: State
Num.Obs.	2163687	2163687	2163686	2163846	2163733
R2	0.122	0.106	0.112	0.119	0.120

NOTES: Estimates are presented as odds ratios (95% CI) and are the result of logistic regression predicting MFM involvement in care. Variable reference categories are denoted by "ref." Rural or urban location was determined by the Rural Urban Commuting Area (RUCA) for the person's ZIP code. We categorized RUCA codes into 2 standard categories: urban (codes 1.0, 1.1, 2.0, 2.1, 3.0, 4.1, 5.1, 7.1, 8.1, and 10.1), and rural (4.0, 4.2, 5.0, 5.2, 6.0, 6.1, 7.0, 7.2, 7.3, 7.4, 8.0, 8.2, 8.3, 8.4, 9.0, 9.1, 9.2, 10.0, 10.2, 10.3, 10.4, 10.5, and 10.6).[7] SVI refers to models in which the four themes of the CDC's Social Vulnerability Index are included as controls. ICE Quintiles refer to models where Index of Concentration at the Extremes (ICE) measures for race and income were included as controls, as calculated in eMethods 2. Other Covariates included in all models were age at delivery, number of sample pregnancies, and delivery year.

© 2025 Sullivan HK et al. *JAMA Network Open*.

eFigure 1: Unadjusted Probability of MFM Involvement in Care of At-Risk Pregnancies by Hospital Service Area (HSA)

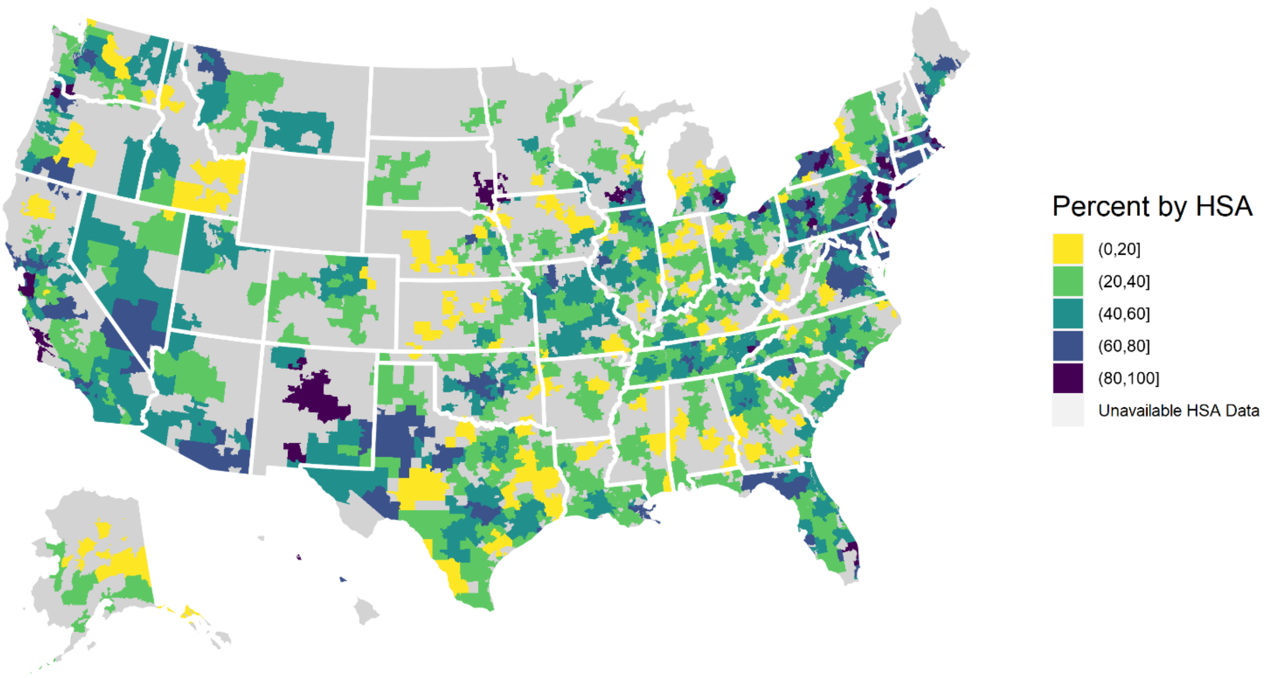

NOTES: This is a map of the United States color coded to show the percent of at-risk pregnancies with MFM service utilization by Hospital Service Area. Map generated from n=1,625,237 pregnancies. HSAs with 10 or fewer pregnancies, or HSAs where there were fewer than 200 MFM claim lines were excluded due to HCCI privacy requirements.

© 2025 Sullivan HK et al. *JAMA Network Open*.

eFigure 2: Adjusted and Unadjusted Probability of MFM Involvement in Care in Full Sample by Hospital Service Area (HSA)

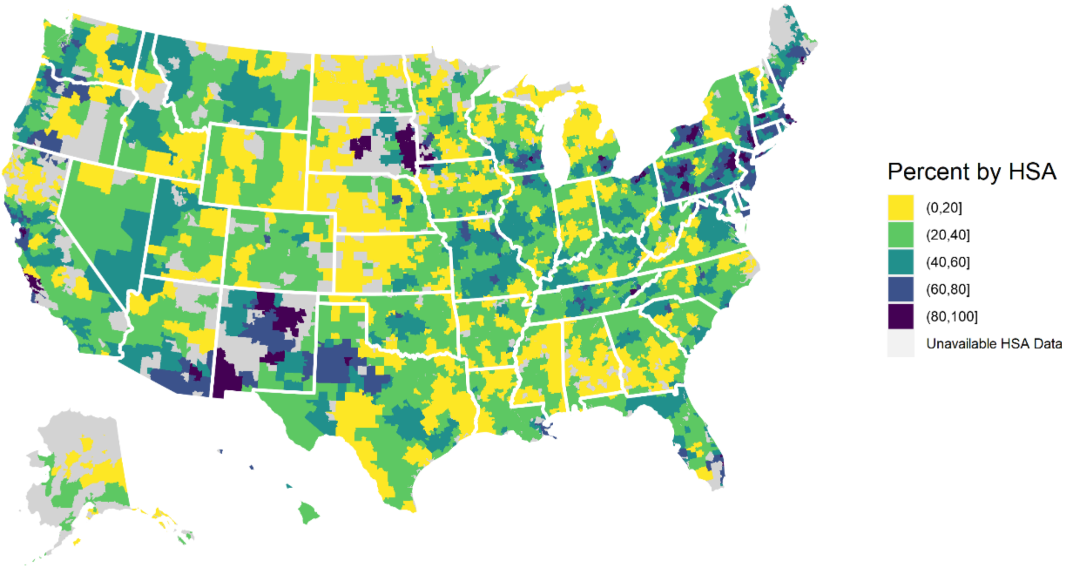

A) Adjusted Probability of MFM Involvement in Care in Full Sample by Hospital Service Area (HSA).

NOTES: This is a map of the United States color coded to show the predicted probability of pregnancies with MFM service utilization by Hospital Service Area. Model estimated on n=2,163,687 pregnancies. Predicted probabilities from a logistic regression adjusted for age, pregnancy risk, driving distance to nearest MFM, delivery year, pregnancy number in sample, and CDC Social Vulnerability Index. Model includes HSA fixed effects, standard errors clustered at the HSA level. HSAs with 10 or fewer pregnancies were excluded due to HCCI privacy requirements.

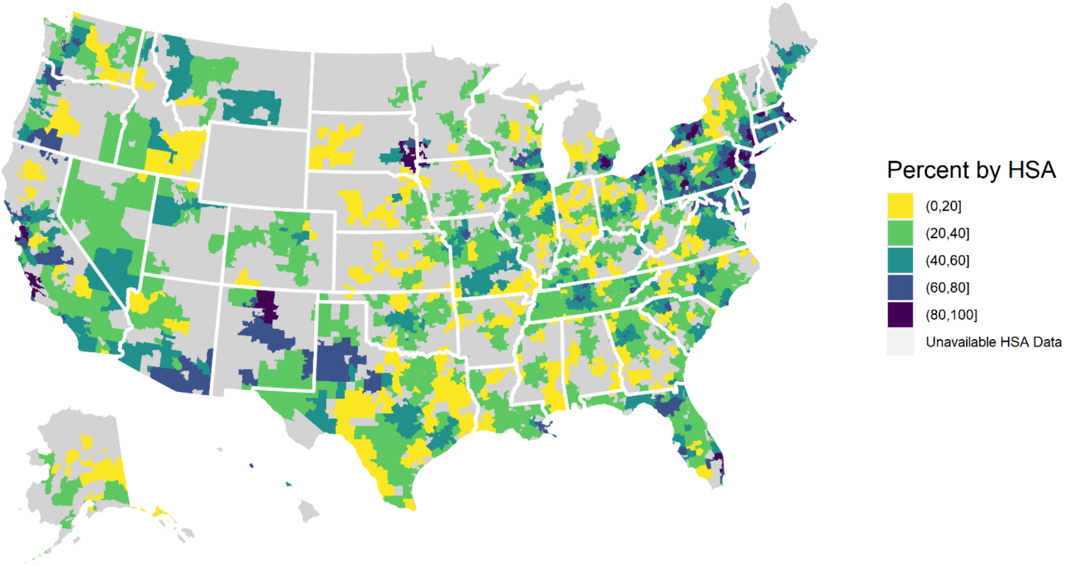

B) Unadjusted Probability of MFM Involvement in Care in Full Sample by Hospital Service Area (HSA).

NOTES: This is a map of the United States color coded to show the percent of pregnancies with MFM service utilization by Hospital Service Area. Map generated from n=2,163,687 pregnancies. HSAs with 10 or fewer pregnancies, or HSAs where there were fewer than 200 MFM claim lines were excluded due to HCCI privacy requirements.

© 2025 Sullivan HK et al. *JAMA Network Open*.

eTable 7: Types of Telemedicine Services Provided by MFMs

**Characteristic**	Pregnancies in sample with any telemedicine MFM service, N = 14,852
Any Ultrasound	398 (2.7%)
Any Evaluation and Management Visit[e]	13,415 (90%)
Any Antenatal Fetal Surveillance	134 (0.9%)
Any Other Service	1,649 (11%)

NOTES: All variables are binary indicators of having a service during pregnancy; as such, numbers will not sum to the total because many pregnancies had more than one telemedicine service. The "other" telemedicine service codes that were found in the largest number of pregnancies were: 96040 (medical genetics and genetic counseling services), 97803 (medical nutrition therapy, follow up), 97802 (medical nutrition therapy, initial visit).

[e] See eTable 2 for ICD-10 codes. This category includes both codes for E&M visits and codes for "telemedicine specific" services.

© 2025 Sullivan HK et al. *JAMA Network Open*.

References

1. Ailes EC, Zhu W, Clark EA, et al. Identification of pregnancies and their outcomes in healthcare claims data, 2008–2019: An algorithm. *PLOS ONE*. 2023;18(4):e0284893. doi:10.1371/journal.pone.0284893

2. Matcho A, Ryan P, Fife D, Gifkins D, Knoll C, Friedman A. Inferring pregnancy episodes and outcomes within a network of observational databases. *PLOS ONE*. 2018;13(2):e0192033. doi:10.1371/journal.pone.0192033

3. MacDonald SC, Cohen JM, Panchaud A, McElrath TF, Huybrechts KF, Hernández-Díaz S. Identifying Pregnancies in Insurance Claims Data: Methods and Application to Retinoid Teratogenic Surveillance. *Pharmacoepidemiol Drug Saf*. 2019;28(9):1211-1221. doi:10.1002/pds.4794

4. American College of Obstetricians and Gynecologists. *OB/GYN Coding Manual: Components of Correct Coding.*; 2023.

5. Hussein M, Waters TM, Chang CF, Bailey JE, Brown LM, Solomon DK. Impact of Medicare Part D on Racial Disparities in Adherence to Cardiovascular Medications Among the Elderly. *Med Care Res Rev*. 2016;73(4):410-436. doi:10.1177/1077558715615297

6. Kozhimannil KB, Hung P, Casey MM, Lorch SA. Factors associated with high-risk rural women giving birth in non-NICU hospital settings. *J Perinatol*. 2016;36(7):510-515. doi:10.1038/jp.2016.8

7. Rural Urban Commuting Area Codes Data. Accessed September 12, 2023. https://depts.washington.edu/uwruca/ruca-uses.php

8. HUD USPS ZIP Code Crosswalk Files | HUD USER. Accessed February 9, 2024. https://www.huduser.gov/portal/datasets/usps_crosswalk.html

9. Manson S, Schroeder J, Van Riper D, Kugler T, Ruggles S. American Community Survey 2015-2019. Published online 2022. doi:http://doi.org/10.18128/D050.V17.0

10. Krieger N, Waterman PD, Spasojevic J, Li W, Maduro G, Van Wye G. Public Health Monitoring of Privilege and Deprivation With the Index of Concentration at the Extremes. *Am J Public Health*. 2016;106(2):256-263. doi:10.2105/AJPH.2015.302955

11. ZIP Code Distance Database: 2016 Centroid File. NBER. Accessed June 22, 2023. https://www.nber.org/research/data/zip-code-distance-database

12. Vilimpoc M. Google Sheets Geocoding Macro. Published online August 3, 2023. Accessed August 14, 2023. https://github.com/nuket/google-sheets-geocoding-macro

13. Build apps with HERE Maps API and SDK Platform Access | HERE Developer. Accessed October 14, 2023. https://developer.here.com

14. Eberth JM, Hung P, Benavidez GA, et al. The Problem Of The Color Line: Spatial Access To Hospital Services For Minoritized Racial And Ethnic Groups. *Health Affairs*. 2022;41(2):237-246. doi:10.1377/hlthaff.2021.01409

15. Murrin S. State Standards for Access to Care in Medicaid Managed Care. *Office of the Inspector General*. Published online 2014.

16. Indications for Outpatient Antenatal Fetal Surveillance. Accessed October 30, 2023. https://www.acog.org/clinical/clinical-guidance/committee-opinion/articles/2021/06/indications-for-outpatient-antenatal-fetal-surveillance

© 2025 Sullivan HK et al. *JAMA Network Open*.

17. Availability of Outpatient Maternal Fetal Medicine and Specialty Care for Women With High Risk Pregnancies | Agency for Healthcare Research and Quality. Accessed October 17, 2024. https://www.ahrq.gov/pqmp/measures/outpatient-speciality-care.html

18. U.S. Department of Agriculture, Economic Research Service. Documentation: 2010 Frontier and Remote (FAR) Area Codes. Accessed October 30, 2024. https://www.ers.usda.gov/data-products/frontier-and-remote-area-codes/documentation/

© 2025 Sullivan HK et al. *JAMA Network Open*.
