# Supplementary material for: Use of Maternal-Fetal Medicine Subspecialist Services by Commercially Insured Pregnant People
Source: JAMA Netw Open. 2025 Jan 13;8(1):e2454565. doi: 10.1001/jamanetworkopen.2024.54565 (PMC11731212; doi:10.1001/jamanetworkopen.2024.54565)
Supplement: Supplement 2. — Data Sharing Statement [file jamanetwopen-e2454565-s002.pdf]

Data Sharing Statement

Sullivan. Use of Maternal-Fetal Medicine Subspecialist Services by Commercially Insured Pregnant People. *JAMA Netw Open*. Published January 13, 2025.
doi:10.1001/jamanetworkopen.2024.54565

Data
Data available: No

Additional Information
Explanation for why data not available: The data was provided by the Health Care Cost Institute. The Data Use Agreement does not allow us to directly share the data due to patient confidentiality.
